# Imaging endogenous macrophage iron deposits reveals a metabolic biomarker of polarized tumor macrophage infiltration and response to CSF1R breast cancer immunotherapy

**DOI:** 10.1038/s41598-018-37408-7

**Published:** 2019-01-29

**Authors:** Avigdor Leftin, Nir Ben-Chetrit, Johanna A. Joyce, Jason A. Koutcher

**Affiliations:** 1 Department of Medical Physics, Memorial Sloan Kettering Cancer Center, New York, NY 10065 USA; 2 Cancer Biology and Genetics Program, Memorial Sloan Kettering Cancer Center, New York, NY 10065 USA; 3 Department of Medicine, Memorial Sloan Kettering Cancer Center, New York, NY 10065 USA; 4 Present Address: Department of Medicine, Weill-Cornell Medical College, New York, NY 10021 USA; 5 Present Address: Department of Oncology, Ludwig Institute of Cancer Research, University of Lausanne, CH-1066 Lausanne, Switzerland

## Abstract

Iron deposits are a phenotypic trait of tumor-associated macrophages (TAMs). Histological iron imaging and contrast-agent free magnetic resonance imaging (MRI) can detect these deposits, but their presence  in human cancer, and correlation with immunotherapeutic response is largely untested. Here, primarily using these iron imaging approaches, we evaluated the spatial distribution of polarized macrophage populations containing high endogenous levels of iron in preclinical murine models and human breast cancer, and used them as metabolic biomarkers to correlate TAM infiltration with response to immunotherapy in preclinical trials. Macrophage-targeted inhibition of the colony stimulating factor 1 receptor (CSF1R) by immunotherapy was confirmed to inhibit macrophage accumulation and slow mammary tumor growth in mouse models while also reducing hemosiderin iron-laden TAM accumulation as measured by both iron histology and *in vivo* iron MRI (FeMRI). Spatial profiling of TAM iron deposit infiltration defined regions of maximal accumulation and response to the CSF1R inhibitor, and revealed differences between microenvironments of human cancer according to levels of polarized macrophage iron accumulation in stromal margins. We therefore demonstrate that iron deposition serves as an endogenous metabolic imaging biomarker of TAM infiltration in breast cancer that has high translational potential for evaluation of immunotherapeutic response.

## Introduction

In most cancers, macrophage infiltration is linked to negative clinical outcomes such as poor survival, metastatic dissemination, and evasion of anti-tumor immune mechanisms^1–4^. Major efforts are underway to understand the function of macrophage infiltrates in the tumor microenvironment in order to develop new treatments such as immunotherapies that target macrophages and inhibit these deleterious outcomes. To support these efforts, there is an increasing need for macrophage biomarkers and imaging approaches that allow for the localization of the targeted macrophage populations according to metabolic phenotype or function and measurement of their response to therapy. Histological methods are useful for quantification of macrophage behavior, but *in vivo* characterization is not possible, and definition of specific phenotypic properties such as polarization status or metabolism can be difficult to generalize from selective biopsy due to tissue intrinsic aspects of macrophage function and the heterogeneous nature of the tumor microenvironment^5,6^. *In vivo* approaches such as positron emission tomography (PET) can provide information about tumor macrophage presence, but repeated imaging is limited due to the accumulation of radioactive dose, and resolution of infiltrating macrophages is also limited by current technology^7,8^. As an *in vivo* imaging tool, magnetic resonance imaging (MRI) can be used to map many metabolic pathways associated with cancer including glycolysis^9,10^, the tricarboxylic acid cycle^11^, phospholipid and ATP metabolism^12,13^, dependencies on perfusion and hypoxia^14^, pH^15^, and oxidation/reduction balance^16^. Despite this arsenal of anatomical and functional molecular protocols, these non-invasive approaches are usually not able to resolve and assign spatial differences in metabolism to specific immune cell populations within the tumor. This is because the metabolic properties of these populations are often obscured as they share similar metabolic pathways to the cancer cells, have smaller relative population sizes, and more heterogeneous spatial distributions compared to the bulk of the tumor^17^. Given the available resolution of most metabolic MRI techniques this leads to an average representation of the spatial distribution of metabolites, often reflecting just the dominant cellular population, i.e. the cancer cells, in the metabolic images.

In order to enable the imaging of macrophages according to their metabolic status, we sought to identify metabolic pathways that exhibit higher specificity for these populations rather than cancer cells or other cellular species. Iron metabolism, the processes by which uptake, storage, and re-export of iron takes place, is conserved in most mammalian cells^18^. However, macrophages in particular are known to play a central role in systemic homeostasis of iron according to their unique genetic program that enables them to handle high metabolic flux of this micronutrient systemically and in the tumor microenvironment^19–21^. In this iron-regulating role, macrophages can exhibit a unique phenotypic trait, namely the accumulation of aggregates comprised of iron known as hemosiderin^22^. Recently, we identified endogenous hemosiderin iron deposition as a putative pan-tissue biomarker of TAMs by using clinical iron-sensitive MRI methods (FeMRI) and Prussian blue iron histology without contrast agents to detect accumulated iron in hemosiderin-laden macrophages (HLMs) of murine prostate, breast, and metastatic cancer models^23,24^. Technically, high-iron concentration FeMRI pixel regions and Prussian blue positive regions indicate the location of macrophage iron deposits that sets them apart from other lower concentration bio-iron sources such as blood due to the physical magnetic and chemical properties of the solid iron stores^25–33^. Similar high-resolution MRI and histological iron imaging approaches can also be used to identify macrophage targets in cancer but traditionally require intravenous injections of iron nanoparticle contrast agents that rely on macrophage phagocytosis rather than metabolism in a manner similar to many PET probes^34,35^. However, caveats of the nanoparticle-enhanced MRI and histological techniques include off-target delivery following from the enhanced permeability and retention effect contributed by highly vascularized leaky tumors that reduces specificity for the macrophage deposits^36^, and the nanoparticles themselves can induce polarization of macrophages that can potentially bias the metabolic function and the therapeutic response of the targeted populations^37–39^. By recognizing the tendency of macrophages to metabolically accumulate hemosiderin—which generates high-iron contrast akin to that produced using iron nanoparticle injections^40^ —microscopic deposits of these cells can be quantified in terms of their abundance and spatial distribution by MRI and histology without contrast agents according to their innate iron metabolism. While these prior studies associated HLMs with TAMs and therefore suggest that they can be used as probes of TAM infiltration to gauge efficacy of immune therapy, here we define the spatial correlations of these metabolically-unique TAM infiltrates with immunotherapy response, and prospectively characterize their distribution in human breast cancer using histological iron imaging in order to support the translation of such combination metabolic iron imaging and therapy approaches to the clinic.

## Results

### Spatial profiling of tumor macrophage iron deposits with iron imaging

**Figure 1:**
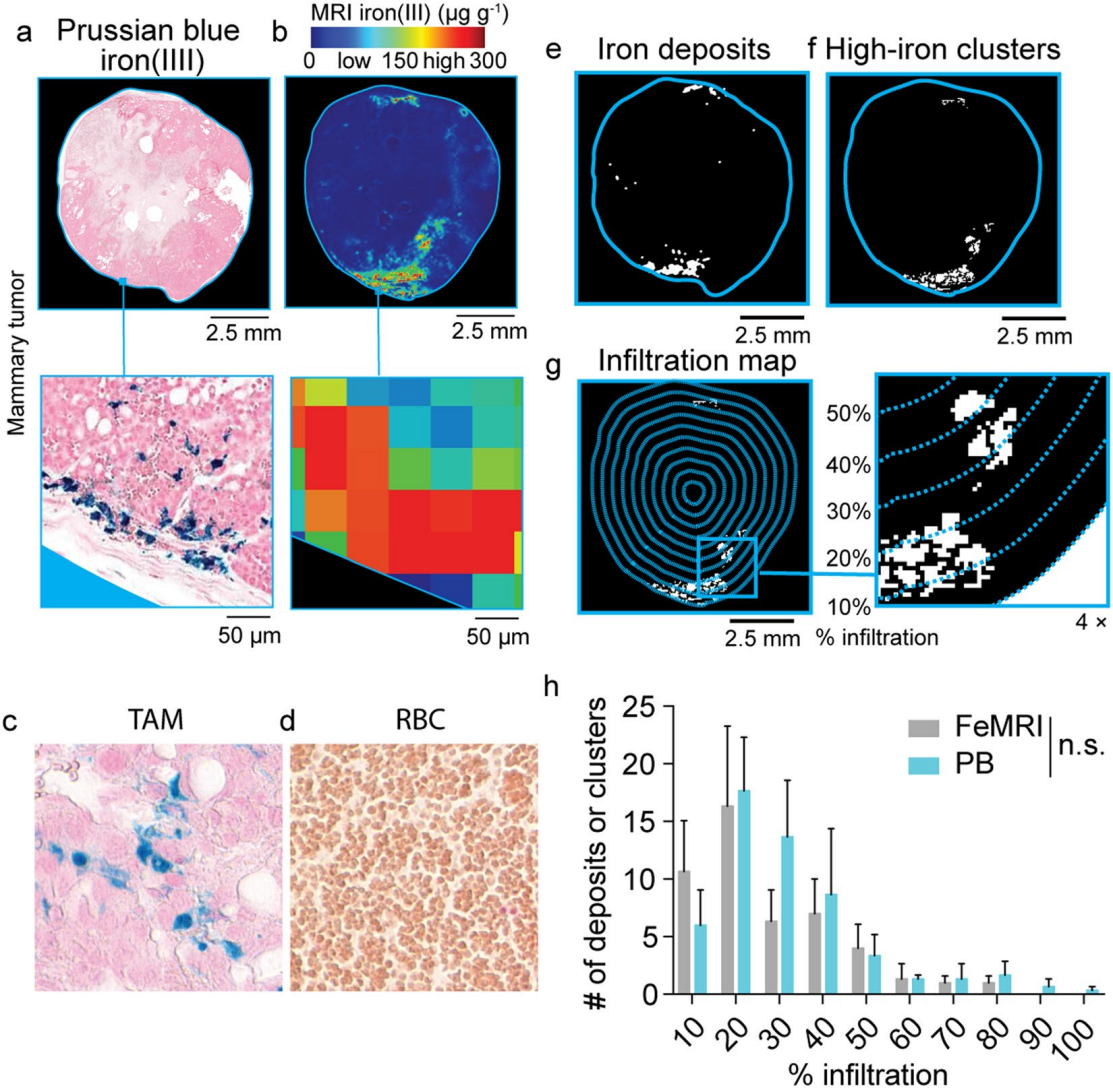
Imaging and spatial profiling of tumor macrophage iron deposits *ex vivo* with iron MRI (FeMRI) and Prussian blue iron histology. (**a**) Representative Prussian blue iron histology of MMTV-PyMT TS1 mammary tumor cross-section. Scale bar 2.5 mm. Expansion shows iron^+^ macrophage deposits. Expansion scale bar 50 μm. (**b**) Representative *ex vivo* FeMRI of MMTV-PyMT TS1 mammary tumor cross-section. Scale bar 2.5 mm. Expansion shows high-iron pixel clusters. Expansion scale bar 50 μm. (**c**) Representative Prussian Blue iron histology of tumor associated macrophages (TAM), and (**d**) red blood cells (RBC). Note iron^+^ macrophages and iron^−^ RBCs. (**e**) Iron deposit mask from Prussian blue histology. Scale bar 2.5 mm. (**f**) High-iron FeMRI cluster mask. Scale bar 2.5 mm. (**g**) Infiltration mapping using radial decile region rake sampling overlaid on high-iron MRI cluster mask. Scale bar 2.5 mm. 4x expansion shows high-iron FeMRI clusters and decile boundaries. (**h**) Infiltration profile showing number of histological iron deposits from Prussian blue (PB) and high-iron FeMRI pixel clusters (FeMRI) as a function of percent (%) infiltration into the MMTV-PyMT TS1 mammary tumors. (mean + s.e.m. n = 3 tissue cross-sections, n.s. p > 0.05 Kolmogorov-Smirnov test).

Endogenous FeMRI methods are increasingly favored over the use of invasive biopsy using Prussian blue iron imaging for measurement of non-heme iron concentrations in liver, heart, and brain^26–28,41–44^. While these methods mitigate the sampling bias introduced by selective biopsy, they conventionally rely on whole organ averages of cellular iron loading, and therefore neglect spatial heterogeneity indicative of TAM infiltration. We rationalized that we could also use FeMRI and correlative Prussian blue histology to detect, resolve, and quantify the spatial distributions of localized TAM iron deposits in breast cancer tumors by addressing spatial heterogeneity of cellular iron deposits through utilization of image analysis algorithms that enable the automatic detection, quantification, and localized mapping of HLM deposits in the iron images^40^. As proof-of-concept, we directly compared histological sections stained for iron using Prussian blue that is specific for HLMs (Fig. 1a), with iron maps generated by *ex vivo* FeMRI-microscopy (Fig. 1b) of co-registered tissue sections obtained from an orthotopic TS1 breast cancer model used commonly in TAM research whose tumors are promoted under control of the murine mammary tumor virus which drives expression of the mammary gland specific polyoma virus middle T-antigen (MMTV-PyMT)^45–49^. Iron^+^ TAMs were determined to be the dominant species generating distinct high-iron pixel clusters in the MMTV-PyMT tumor cross-sections as comparison of Prussian Blue stained macrophage (Fig. 1c) and red blood cells (Fig. 1d), another candidate for contributing to iron contrast because of their heme cargo, showed they do not stain for Prussian blue iron and thus contribute only to low FeMRI contrast, further corroborating the specificity of the method for HLMs^23^. We then interrogated the spatial distribution of the HLMs in the histological and FeMRI iron maps to compare these measurements as a score of TAM infiltration. Analysis of the histological (Fig. 1e), and MRI images (Fig. 1f) for high concentrations of iron yielded maps of the iron containing TAMs. The histological iron deposits and FeMRI pixel clusters were then graphed as a function of position in the tumor (% infiltration, Fig. 1g), and the radial infiltration profiles of the histological deposits and MRI clusters were found to be the same (Fig. 1h, p > 0.05). This confirmed the equivalence between the HLM measurements by FeMRI and histology, and further provided a novel means to map the spatial distribution of the HLM deposits according to metabolic status with cellular sensitivity.

### Correlation between immunotherapeutic CSF1R inhibitor response and polarized iron deposit accumulation

**Figure 2:**
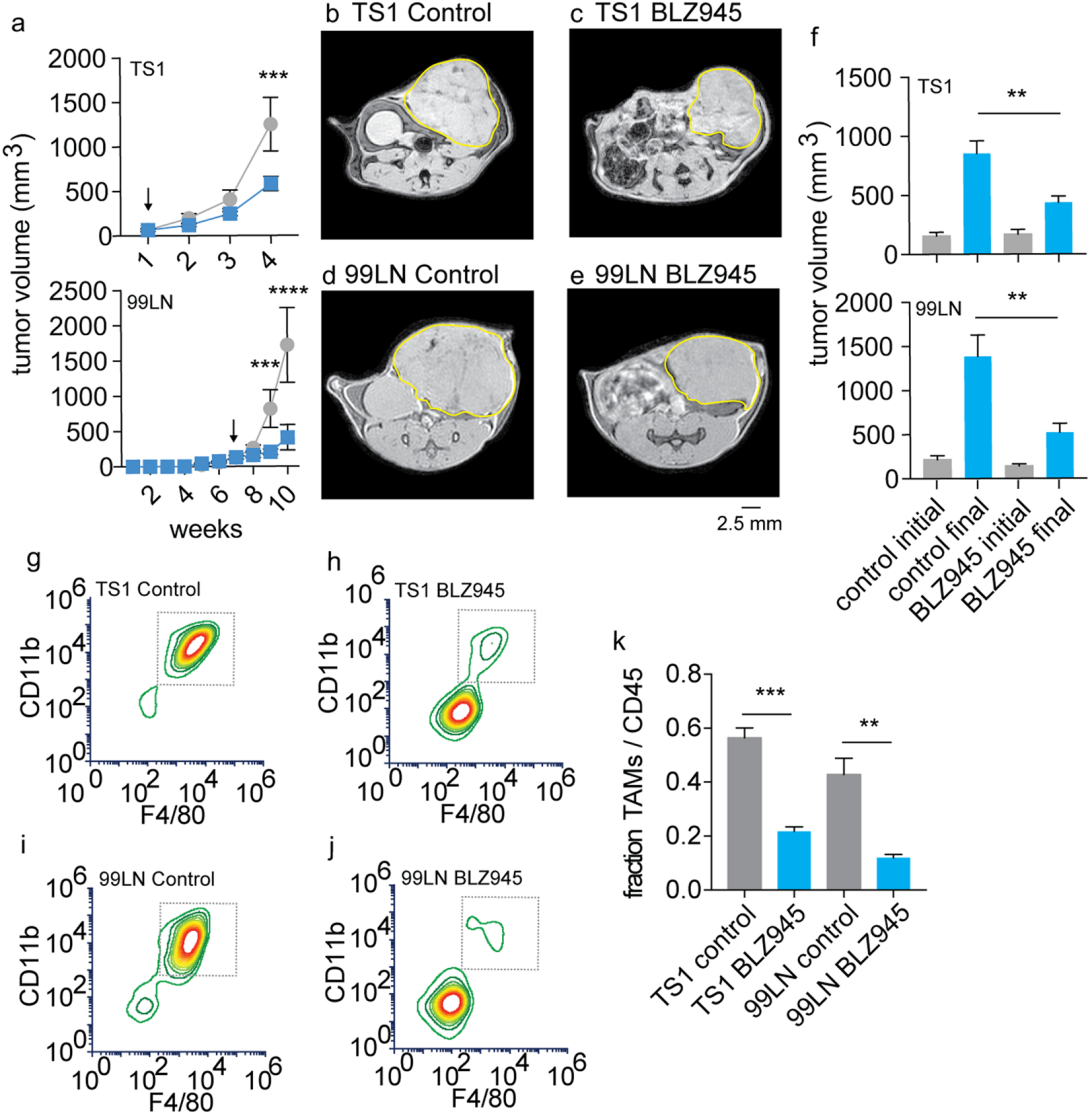
Validation of CSF1R immunotherapy effects on tumor growth and TAM accumulation in PyMT-MMTV breast cancer models. (**a**) Murine breast tumor models were established by orthotopic mammary fat pad injections and measured by caliper in TS1 and 99LN models during administration of the CSF1R inhibitor BLZ945 (200 mg/kg). Arrows indicate start of BLZ945 treatment (mean ± s.e.m. n = 5 mice/group, ***p < 0.001, ****p < 0.0001, 2-way ANOVA with Tukey’s multiple comparison test). Representative first-echo images from MGE MRI experiments made at study endpoints in control and BLZ945 treated (**b**,**c**) TS1 and (**d**,**e**) 99LN MMTV-PyMT models. (**f**) Pre-treatment and endpoint tumor volumes measured by MRI in the CSF1R inhibitor trials. (mean + s.e.m., n = 8 mice/group, **p < 0.01, two-tailed unpaired students t-test). Flow cytometry panels of TAMs (live CD45^+^Ly6c^−^Ly6g^−^ cells gated on CD11b^+^F4/80^+^ cells) obtained from control and BLZ945 treated (**g**,**h**) TS1, and (**i**,**j**) 99LN tumors. (**k**) TAM frequency with respect to total CD45^+^ myeloid cells in the TS1 and 99LN CSF1R inhibitor trials (mean + s.e.m. n = 4 mice/group, **p < 0.01, ***P < 0.001, two-tailed unpaired students t-test).

To then further establish these TAM iron deposits as immunotherapy targets, we initiated preclinical CSF1R (colony-stimulating factor-1 receptor) inhibitor (BLZ945) trials in murine breast cancer models. This receptor kinase inhibitor blocks the interaction between the cytokine colony stimulating factor 1 (CSF1) that mediates macrophage accumulation in tumors via signaling with its receptor CSF1R^50,51^. The drug has been shown to have the primary immunological effect of inhibiting the accumulation of TAMs in tumors, making it an excellent candidate for testing the iron imaging approaches, and it has the coincident therapeutic effect of slowing the growth of some breast, cervical, brain, and other cancers^48,52–55^. Cell-line derived TS1 and 99LN MMTV-PyMT tumors orthotopically implanted in the mammary fat pads of their respective syngeneic FVB/N and C57BL6 hosts were studied. Treatment with the small molecule CSF1R inhibitor BLZ945 was initiated when tumors reached approximately 100 mm^3^ in the TS1 and 99LN models. Treatment continued until control tumors reached or exceeded 1 cm^3^ measured by caliper to establish pre-treatment and endpoint imaging time points, and significant tumor growth inhibition was observed in both models with CSF1R inhibition by these endpoints (Fig. 2a, p < 0.001–p < 0.0001). Subsequent measurement of the tumor volumes made by MRI in the imaging studies of the control and BLZ945 treated TS1 (Fig. 2b,c) and 99LN models (Fig. 2d,e) recapitulated the reductions in tumor volumes established by the initial pilot trials where TS1 tumor volumes were 51% of controls, and the drug also limited the growth of the 99LN model tumors to approximately 34% of the untreated groups (Fig. 2f, p < 0.01).

To then characterize the primary immunological effect of the CSF1R inhibitor on TAM accumulation as has been done in previous studies in MMTV-PyMT models with the BLZ945 drug and others^45,48^, whole tumors from the TS1 and 99LN models were collected at imaging endpoints and single-cell suspensions were prepared from the homogenates. Fluorescence staining of the cells for live CD45^+^Ly6c^−^Ly6g^−^CD11b^+^F4/80^+^ TAMs was then performed and the frequency of these cells was quantified by flow cytometry. Treatment with BLZ945 reduced the frequency of TS1 TAMs (Fig. 2g,h), and similarly 99LN models also exhibited reductions in TAMs (Fig. 2i,j) in accord with the previous studies of the inhibitor, thereby providing further preclinical validation for our imaging studies. Overall the CSF1R immune therapy lowered the frequency of TAMs with respect to total CD45^+^ cells in the tumors significantly with levels of BLZ945 treated TAM fraction by approximately 30% of the control levels in both TS1 and 99LN groups (Fig. 2k, p < 0.001– p < 0.01).

**Figure 3:**
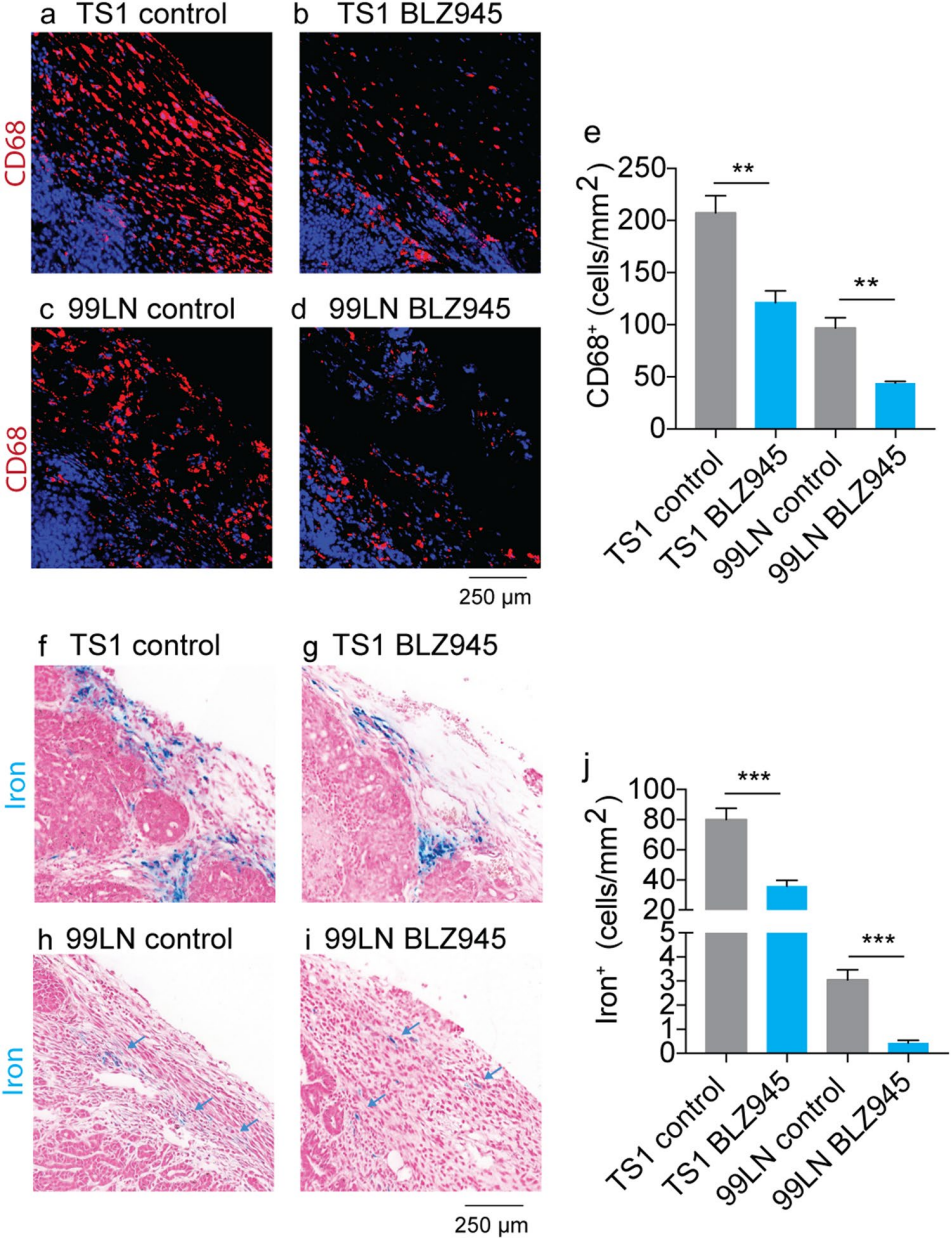
CD68 immunofluorescence and Prussian blue imaging of macrophage iron deposits in MMTV-PyMT murine breast cancer models of CSF1R immunotherapy. Representative CD68 macrophage immunofluoresent imaging in control and BLZ945 treated (**a**,**b**) TS1, and (**c,d**) 99LN tumors. Scale bar 250 µm (**e**) Absolute counts of CD68^+^ macrophages per mm^2^ MMTV-PyMT tumor cross-section in BLZ945 CSF1R inhibitor trials (mean + s.e.m. n = 4 mice/group, **p < 0.01, two-tailed unpaired students t-test). Representative iron staining using Prussian blue iron histochemistry in control and BLZ945 treated MMTV-PyMT (**f**,**g**) TS1, and (**h**,**i**) 99LN tumors. Scale bar 250 µm (**j**) Absolute counts of iron^+^ macrophages per mm^2^ tumor cross-section in the CSF1R inhibitor trials (mean + s.e.m. n = 4 mice/group, ***p < 0.001, two-tailed unpaired students t-test).

In order to quantify the response of iron^+^ TAMs to the BLZ945 inhibitor, we performed Prussian blue histology specific for the TAM iron deposits beside CD68 macrophage histology as a general marker of TAMs. First, digitized images of paraffin-embedded whole axial cross-sections of the tumor collected at CSF1R trial endpoints were analyzed by counting all TAMs according to their CD68 staining to measure general response to the CSF1R inhibitor. Reduced numbers of CD68^+^ infiltrating macrophages were found in both the TS1 (Fig. 3a,b) and 99LN cohorts (Fig. 3c,d) confirming previous studies of this inhibitors effects on TAM accumulation in MMTV-PyMT models^45^. Overall, CD68^+^ macrophages were lower by approximately 42% in the TS1 model and 55% in the 99LN model following BLZ945 treatment (Fig. 3e, p < 0.01) generally recapitulating the reductions in TAMs measured by flow cytometry in the trials. Histological assessments of HLMs using Prussian blue iron staining were performed in the same manner as CD68 histology. TS1 tumors in the syngeneic FVB/N background exhibited numerous HLM deposits consisting of colonies of iron^+^ TAMs found largely in stromal margins of the paraffin embedded tumor cross-sections (Fig. 3f), and treatment with BLZ945 reduced the number of these cellular species (Fig. 3g). The iron^+^ TAMs were relatively fewer in the 99LN models, but were still detected in the digital image analysis of the Prussian blue iron-stained 99LN tumors (Fig. 3h), and these iron deposits were also lowered with BLZ945 treatment (Fig. 3i). Administration of the CSF1R inhibitor reduced iron^+^ TAM accumulation by approximately 50% in the TS1 models and 85% in the 99LN models (Fig. 3j, p < 0.001), again corroborating the primary effect of the CSF1R inhibitor on TAM accumulation, and further indicating that this drug also effects iron containing TAM populations.

**Figure 4:**
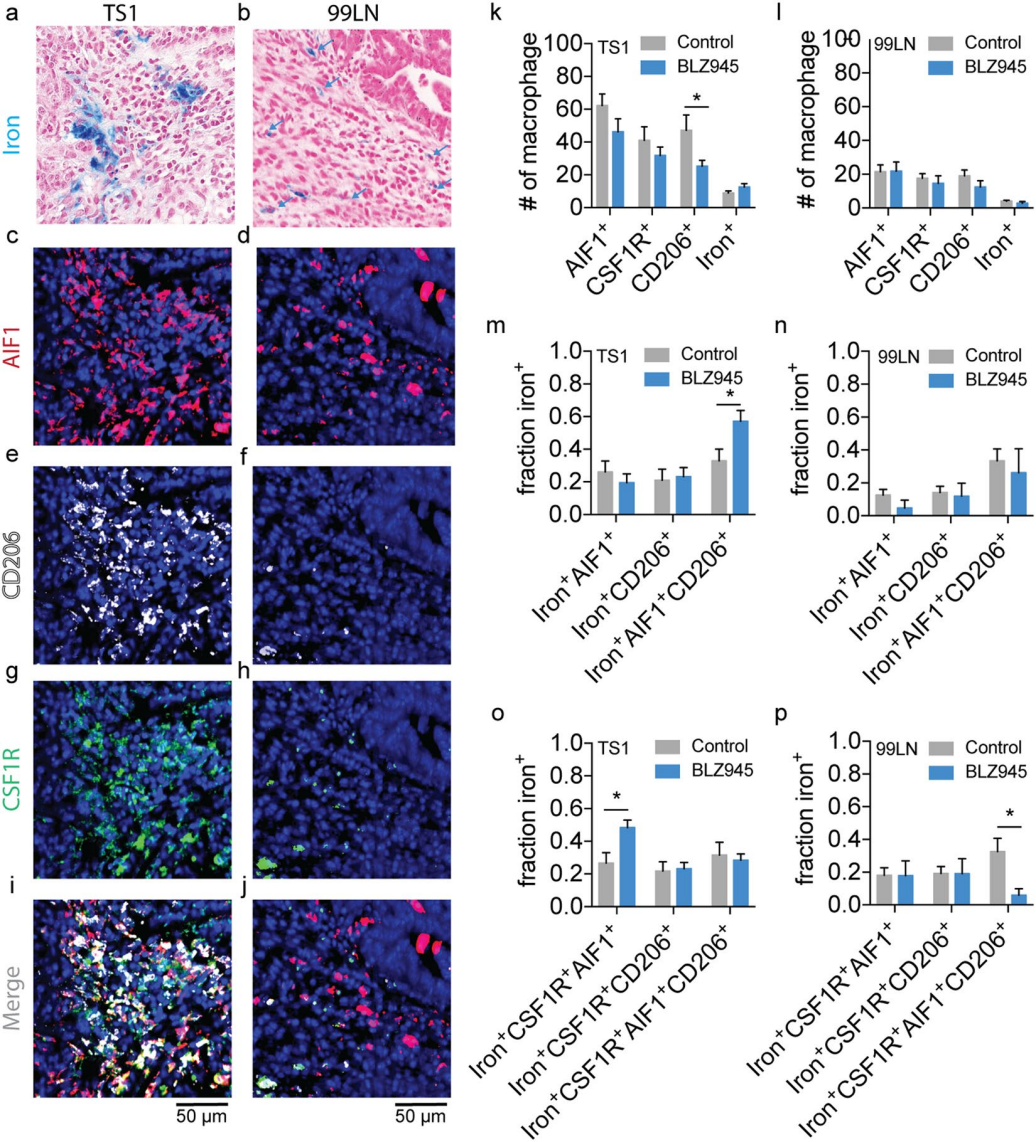
Immunofluorescent and Prussian blue imaging of macrophage iron deposit polarization and CSF1R status in MMTV-PyMT murine breast cancer models of CSF1R immunotherapy. Representative registered fields centered on TAM iron deposits in MMTV-PyMT TS1 and 99LN tumors stained for iron with Prussian blue (**a**,**b**), allograft inflammatory factor-1 (AIF1, M1-like, **c**,**d**), mannose receptor (CD206, M2-like, **e**,**f**), colony stimulating factor 1 receptor (CSF1R, **g**,**h**) and the combined immunofluorescent channels(**i**,**j**). Note fields of control tumors are shown, but are representative of both control and BLZ945 treated groups. Each field is 200 µm × 200 µm. Scale bar 50 µm. Blue arrows indicate location of iron^+^ macrophage in 99LN field. Number (#) of AIF1^+^, CSF1R^+^, CD206^+^ and iron^+^ macrophages detected per field for control and BLZ945 treated (**k**) TS1 and (**l**) 99LN MMTV-PyMT mammary tumor models (mean + s.e.m. n = 20 fields for TS1 control and BLZ945, n = 20 fields for 99LN control and n = 7 fields for 99LN BLZ945, *p < 0.05, Mann-Whitney test). Fraction of total iron^+^AIF1^+^, iron^+^CD206^+^, and iron^+^AIF1^+^CD206^+^ macrophages detected per field in control and BLZ945 treated groups for (**m**) TS1 and (**n**) 99LN mammary tumor models. Fraction of total iron^+^CSF1R^+^AIF1^+^, iron^+^CSF1R^+^CD206^+^ and iron^+^CSF1R^+^AIF1^+^CD206^+^ macrophages detected per field in control and BLZ945 treated groups for (**o**) TS1 and (**p**) 99LN mammary tumor models (mean + s.e.m. n = 20 fields for TS1 control, n = 20 fields TS1 BLZ945, n = 20 fields for 99LN control and n = 7 fields for 99LN BLZ945, *p < 0.05, Mann-Whitney test).

Macrophage accumulation in tumors fulfills both inflammatory and anti-inflammatory roles, but little is known about the polarization and CSF1R status of iron^+^ TAMs. To investigate the polarization status of these macrophage subpopulations, Prussian blue iron-stained histological sections (Fig. 4a,b) were re-stained using multiplexed immunofluorescence for M1-like (Fig. 4c,d, pro-inflammatory, AIF1; allograft inflammatory factor-1), M2-like (Fig. 4e,f, anti-inflammatory, CD206; mannose receptor), and CSF1R receptor (Fig. 4g,h) markers in the TS1 and 99LN models. Controlling for the primary inhibitory effect on the accumulation of iron^+^ populations, fields containing the iron^+^ TAMs in control tumors and fields still containing iron deposits following BLZ945 treatment were identified in the registered tumor cross-sections in order to quantify the co-positivity of the iron^+^ TAMs as a function of M1-like, M2-like, and CSF1R status in these localized regions. Counts of these macrophage populations in the TS1 and 99LN tumors (Fig. 4k,l) showed that TS1 tumors had relatively higher numbers of macrophages expressing polarization markers and CSF1R compared with the 99LN model in accord with the cell counts made independently of polarization status. The CSF1R inhibitor BLZ945 did not greatly effect these general populations, though a small reduction of CD206^+^ macrophage was observed in the TS1 deposit regions (p < 0.05). To specifically assess changes in polarization status of the iron^+^ macrophages with BLZ945 treatment, the fractions of iron^+^ macrophage subpopulations expressing M1-like, M2-like, and mixed M1- and M2-like markers were calculated as a function of the total iron^+^AIF1^+^, iron^+^CD206^+^ and iron^+^AIF1^+^CD206^+^ populations in the TS1 (Fig. 4m) and 99LN models (Fig. 4n). Similarly, the iron^+^AIF1^+^, iron^+^CD206^+^ and iron^+^AIF1^+^CD206^+^ populations were also assessed for CSF1R positivity calculated as a fraction of total iron^+^CSF1R^+^AIF1^+^, iron^+^CSF1R^+^CD206^+^ and iron^+^CSF1R^+^AIF1^+^CD206^+^ macrophages present (Fig. 4o,p). This analysis revealed that while the iron^+^ populations were found co-localized with AIF1 and CD206 polarization markers as well as CSF1R, the fraction of these iron^+^ M1-like and iron^+^ M2-like species and their CSF1R^+^ counterparts in these iron deposit regions were largely unaffected by the CSF1R inhibitor except for small differences in iron^+^AIF1^+^CD206^+^, iron^+^CSF1R^+^AIF1^+^ and iron^+^CSF1R^+^AIF1^+^ CD206^+^ populations in the fields assessed (p < 0.05). This histological analysis indicates that polarization of the iron^+^ populations, and general populations overall is largely unaffected by the CSF1R inhibitor, and also indicated that the HLMs were not significantly biased towards a given polarization state as they were frequently co-localized with multiple markers.

### Iron imaging of macrophage tumor infiltration in CSF1R inhibitor trials

**Figure 5:**
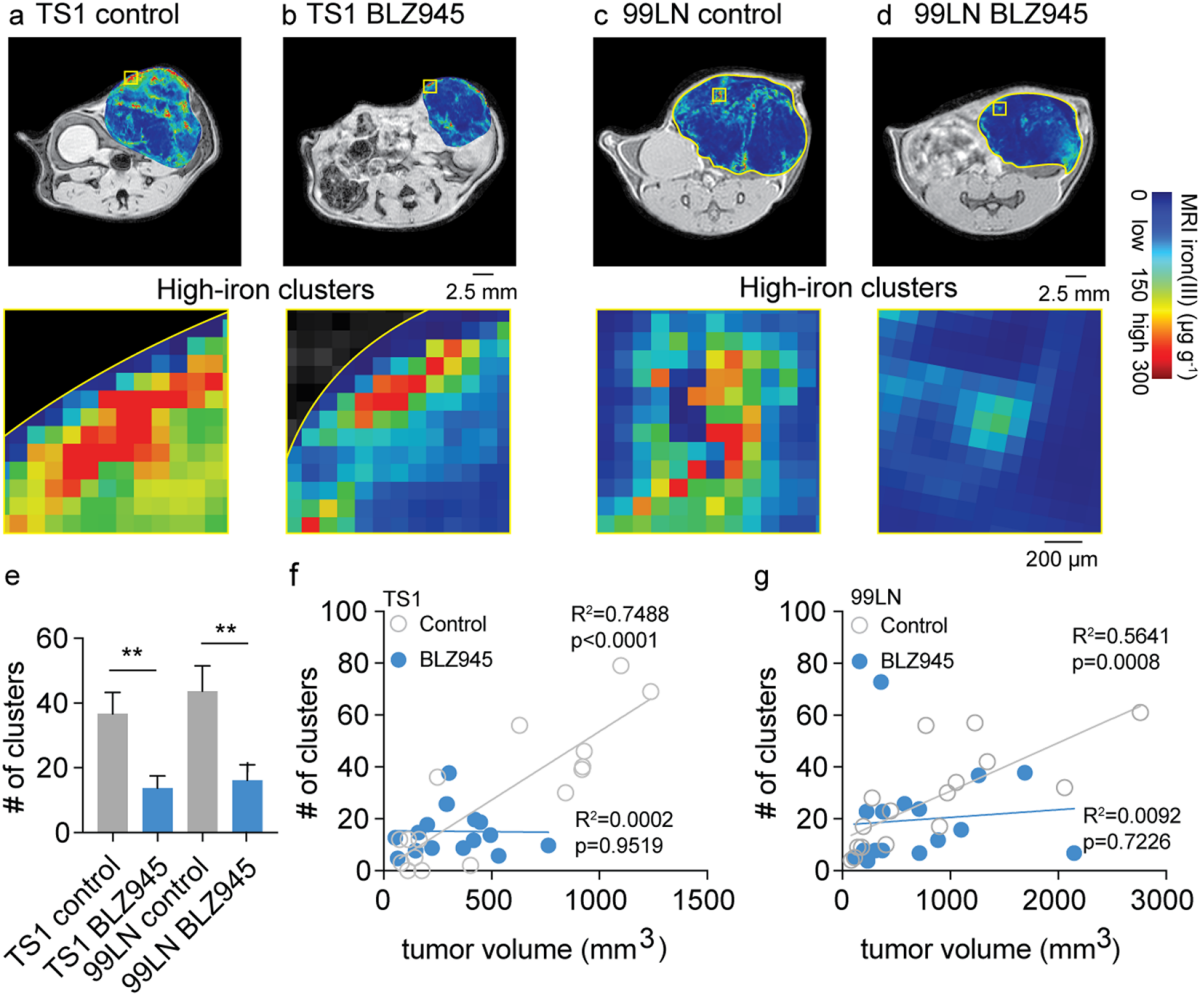
*In vivo* iron MRI (FeMRI) of murine macrophage iron deposits and correlation between immune and therapeutic CSF1R inhibitor response. Representative *in vivo* FeMRI axial cross sections of the mammary tumors are shown in control and BLZ945 treated (**a**,**b**) TS1, and (**c,d**) 99LN models. Scale bar 2.5 mm. Expansions show high-iron pixel clusters. Scale bar 200 µm. (**e**) Number (#) of high-iron FeMRI pixel clusters in the TS1 and 99LN tumors in the CSF1R inhibitor trials (mean + s.e.m. n = 8 mice/group, **p < 0.01 two-tailed unpaired students t-test). Linear correlations between high-iron FeMRI clusters and tumor volumes in the control(ο) and BLZ945-treated(•) (**f**) TS1 and (**g**) 99LN MMTV-PyMT tumor models (n = 8 mice/group, R^2^ and correlation p-value from linear Pearson correlation are shown).

*In vivo* contrast-agent free FeMRI was then used to quantify macrophage iron deposits of the MMTV-PyMT models in the BLZ945 trials, and correlate their detection with the CSF1R inhibitor’s primary immunotherapeutic effects on macrophage accumulation and tumor growth. FeMRI images were quantified using image-processing algorithms demonstrated above in the *ex vivo* analysis. Control and BLZ945 treated MMTV-PyMT mammary tumors exhibited high-iron pixel clusters indicative of macrophage iron deposits in both the TS1 (Fig. 5a,b) and 99LN (Fig. 5c,d) models. High-iron pixel clusters found in BLZ945 treated tumors were approximately 37% of control levels in both models (Fig. 5e p < 0.01) supporting the flow cytometry and histological measurements as shown in Figs 2 and 3. Tumor growth and accumulation of FeMRI clusters were positively correlated in both TS1 (Fig. 5f) and 99LN models (Fig. 5g), and immunotherapeutic response was indicated by a reduction in tumor growth and inhibition of FeMRI cluster accumulation.

**Figure 6:**
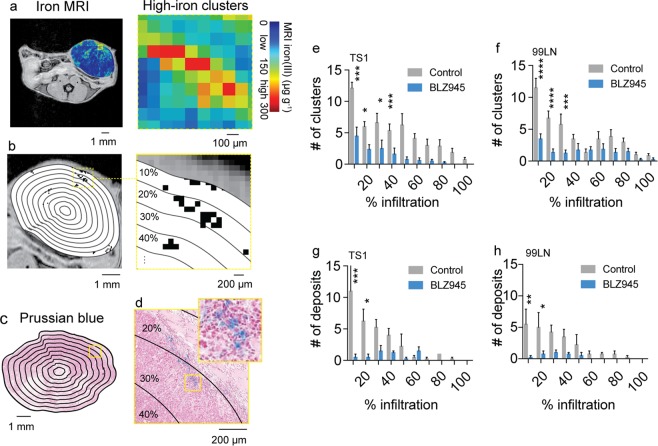
Spatial scores of murine tumor macrophage infiltration in CSF1R immune therapy trials from iron MRI and Prussian blue iron histology. (**a**) *In vivo* iron MRI (FeMRI) of MMTV-PyMT mammary tumor. Scale bar 1 mm. Expansion shows high-iron pixel clusters. Scale bar 100 µm. (**b**) Binary high-iron pixel cluster mask from FeMRI of MMTV-PyMT tumor cross-section. Scale bar 1 mm. Concentric rake region of interest grid overlay used to profile clusters is shown. Expansion shows detected clusters and concentric counting grid. Scale bar 200 µm. (**c**) Prussian blue iron stained cross-section of MMTV-PyMT tumor showing the rake grid overlay used to score the iron deposits. Scale bar 1 mm. (**d**) Expansion showing deposit, i.e. colony of iron^+^ macrophages. Scale bar 200 µm. Infiltration profiles of high-iron clusters from FeMRI in control and BLZ945-treated (**e**) TS1 and (**f**) 99LN MMTV-PyMT models (mean + s.e.m. n = 8 mice/group, *p < 0.05, ***p < 0.001, ****p < 0.0001, 2-way ANOVA with Sidak’s multiple comparison test). Iron^+^ macrophage deposit infiltration profiles from Prussian blue histology in control and BLZ945-treated (**g**) TS1 and (**h**) 99LN MMTV-PyMT models (mean + s.e.m. n = 4 mice/group, *p < 0.05, ***p < 0.001, ****p < 0.0001, 2-way ANOVA with Sidak’s multiple comparison test).

Counts of the high-iron FeMRI pixel clusters and HLM deposits found in histological Prussian blue iron images were also analyzed as a function of position in order to establish spatially-resolved scores of immunotherapeutic response (Fig. 6a–d). The scores of the FeMRI clusters (Fig. 6e,f) and HLM deposits (Fig. 6g,h) both showed higher levels of TAMs at the stromal margins of the tumors with decreasing numbers of iron containing cells found towards the tumor core where less macrophage infiltration generally occurs. Treatment with BLZ945 resulted in overall lower levels of the iron containing regions throughout the tumor cross-sections. The clusters measured by FeMRI and HLM deposits measured by Prussian blue histology were most affected at the outer margins of the tumors by the CSF1R therapy as indicated by the significant reductions observed over these regions (p < 0.05-p < 0.0001). This establishes that contrast-agent free *in vivo* FeMRI can detect and map macrophage iron deposits in a similar manner to *ex vivo* iron histology, and that using FeMRI and iron histology during CSF1R immunotherapy provides measurements of TAM infiltration correlated with the regions of maximal immunotherapeutic response. Thus, iron serves as a novel metabolic biomarker indicating response to immunotherapy treatment that can be monitored *in vivo* using non-invasive MRI technology.

### Prospective survey of polarized macrophage iron deposit infiltration in human breast cancer

**Figure 7:**
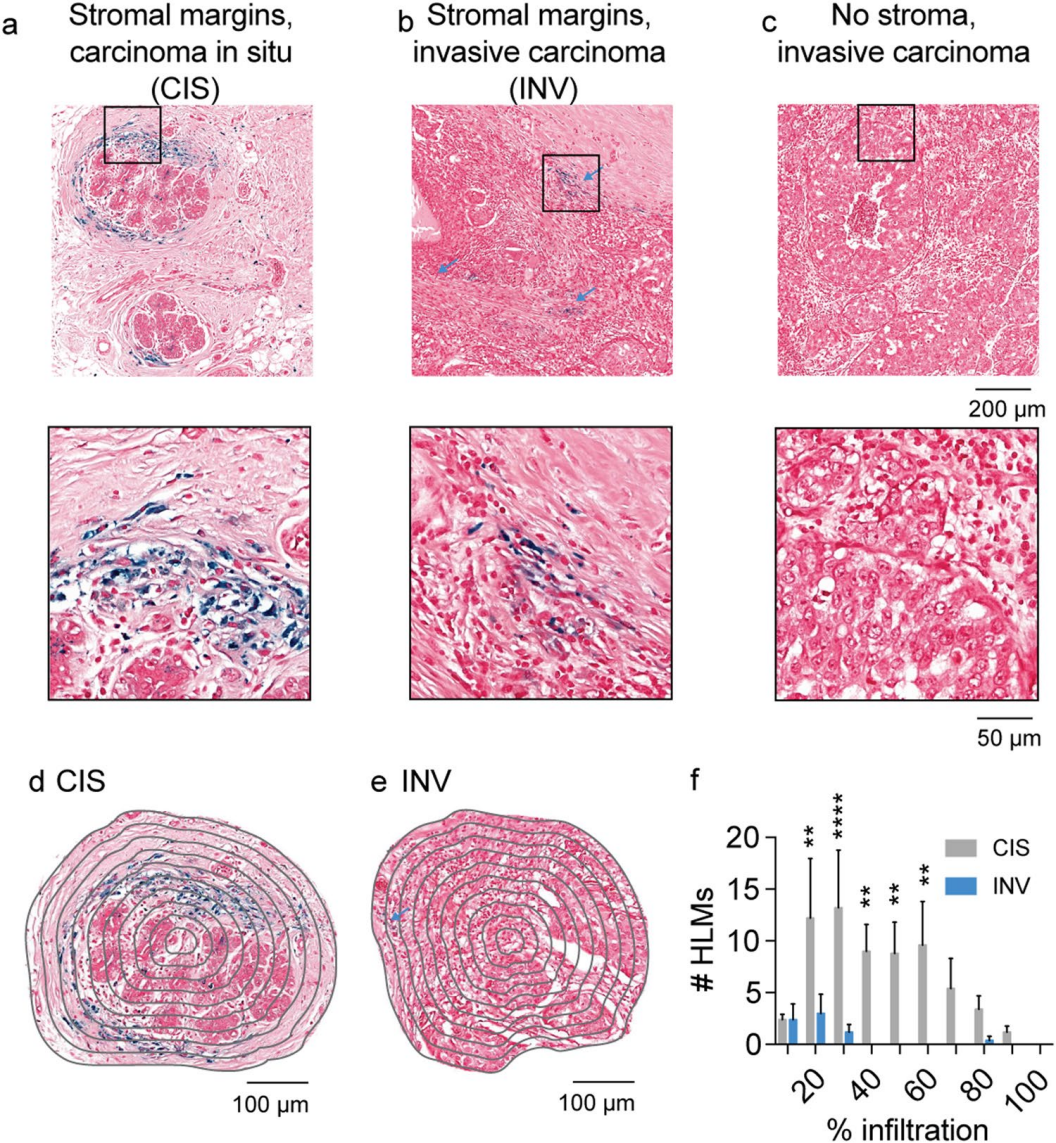
Spatial scores of iron deposits from Prussian blue histology in human breast cancer. Prussian Blue iron histochemistry shows the presence of iron deposits in (**a**) stromal margins of carcinoma *in situ* (CIS) and (**b**) invasive carcinoma (INV, blue arrows). No iron deposits were associated with (**c**) invasive carcinoma exhibiting poorly defined stromal margins. Scale bar 200 µm. Expansions of boxes in (**a**–**c**) shown below. Scale bar 40 µm. Concentric rake region of interest grid overlay used to profile HLMs in (**d**) CIS and (**e**) INV fields. Scale bar 100 µm. (**f**) Iron^+^ macrophage (HLM) infiltration profiles from Prussian blue histology in CIS and INV fields. (mean + s.e.m. n = 5 fields/cancer subtype, **p < 0.01, ****p < 0.0001, 2-way ANOVA with Sidak’s multiple comparison test).

The eventual clinical translation of iron as a metabolic biomarker for macrophage detection and its combination with CSF1R immunotherapy largely depends on whether iron deposits are detectable in human breast cancer. Therefore, prospective surveys of histological samples containing regions of human carcinoma *in situ* and invasive carcinoma were performed by staining paraffin-embedded sections with the Prussian blue method to identify non-heme iron deposits specific to HLMs. Iron deposits were detected in the stromal margins of carcinoma *in situ* (CIS, Fig. 7a), and were also detected at stromal boundaries of invasive carcinoma (INV, Fig. 7b). In regions densely populated by cancer cells in highly invasive carcinoma where stromal margins were not evident these deposits were absent (Fig. 7c). Profiling of HLM deposit infiltration as a function of position across regions indicative of CIS (Fig. 7d) and INV where the HLMS are present (Fig. 7e) further indicated significant spatial differences between the HLMs in these breast cancer microenvironments. Similar to the murine spatial infiltration profiles, human HLMs were more abundant at the outer stromal margins of the tumors, and *in situ* carcinoma was found to exhibit higher numbers of infiltrating HLMs compared with the margins of the invasive carcinoma tumor microenvironments (Fig. 7f, p < 0.01-p < 0.0001). This confirmed the association of macrophage iron deposits with human breast cancer, and shows that while they are commonly found in the tumor-stroma boundaries of both cancer subtypes, they are more prominently observed in the *in situ* pathologies where the stromal margins are better defined compared to invasive carcinoma where such margins can be less evident. As *in situ* carcinoma is thought to precede the emergence of invasive carcinoma, these findings support the translational value of using iron as an early cancer imaging biomarker of TAMs.

**Figure 8:**
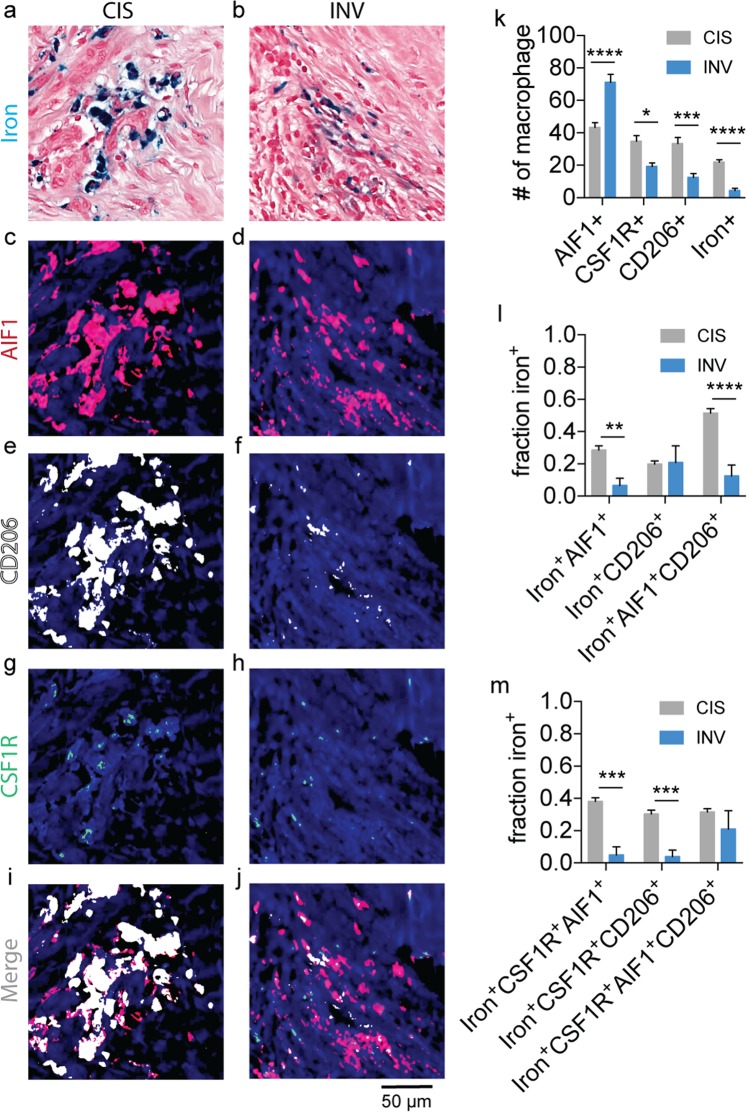
Immunofluorescent and Prussian blue imaging of human macrophage iron deposit polarization and CSF1R status. Representative registered fields of human carcinoma *in situ* (CIS) and invasive carcinoma where iron deposits are found (INV) stained for iron with Prussian blue (**a,b**), allograft inflammatory factor-1 (AIF1, **c,d**), mannose receptor (CD206, **e,f**), colony stimulating factor 1 receptor (CSF1R, **g,h**) and the combined immunofluorescent channels (**i,j**). Each field is 200 µm × 200 µm. Scale bar 50 µm. (**k**) Number (#) of AIF1^+^, CSF1R^+^, CD206^+^ and iron^+^ macrophages detected per CIS and INV field. (mean+s.e.m. n=18 fields for CIS, n=9 fields for INV, *p < 0.05, ***p < 0.001, ****p < 0.0001, Mann-Whitney test). (**l**) Fraction of total iron^+^AIF1^+^, iron^+^CD206^+^, and iron^+^AIF1^+^CD206^+^ macrophages detected per CIS and INV field. (**m**) Fraction of total iron^+^CSF1R^+^AIF1^+^, iron^+^CSF1R^+^CD206^+^, and iron^+^CSF1R^+^AIF1^+^CD206^+^ macrophages detected per CIS and INV field (mean+s.e.m. n=18 fields for CIS, n=10 fields for INV, **p < 0.01, ***p < 0.001, ****p < 0.0001, Mann-Whitney test).

The iron^+^ TAMs in the human cancers were further tested for polarization and CSF1R status using multiplexed immunofluorescence imaging methods as we performed in the murine CSF1R inhibitor trials^56^. The CIS and INV fields stained for Prussian blue iron^+^ HLMs (Fig. 8a,b) were re-stained for inflammatory M1-like macrophages (Fig. 8c,d; AIF1), anti-inflammatory M2-like macrophages (Fig. 8e,f; CD206), and the CSF1R receptor (Fig. 8g,h) in order to determine the co-positivity of iron with these markers (Fig. 8i,j). The total numbers of macrophages were assessed in fields centered on HLM deposits in the stromal margins of the CIS and INV regions. The AIF1^+^, CSF1R^+^, and CD206^+^ macrophages were significantly different in the CIS and INV regions (Fig. 8k) and corresponded to higher numbers of pro-inflammatory AIF1^+^ cells in INV fields (p < 0.0001), while CSF1R^+^ (p < 0.05), CD206^+^ (p < 0.01), and iron^+^ macrophages (p < 0.001) were significantly lower in these same regions. To quantify the association of the iron^+^ macrophages with these markers the fraction of iron^+^AIF1^+^, iron^+^CD206^+^, iron^+^AIF1^+^CD206^+^ macrophage were calculated as a function of the total polarized iron^+^ TAM population (Fig. 8l), and similarly these iron^+^ populations were assessed for co-positivity with the CSF1R marker (Fig. 8m) to determine whether the iron^+^ TAMs in these fields express this receptor to further motivate later immunotherapeutic interventions using CSF1R inhibitors. Overall, significantly higher fractions of iron^+^AIF1^+^ (p < 0.01) and iron^+^AIF1^+^CD206^+^ (p < 0.0001) macrophages were found in CIS microenvironments, while iron^+^CD206^+^ markers were statistically the same in CIS and INV locations (p > 0.05). Additionally, the calculation of the fraction of the iron^+^AIF1^+^ and iron^+^CD206^+^ populations with CSF1R indicated that significantly more iron^+^CSF1R^+^AIF1^+^ (p < 0.001) and iron^+^CSF1R^+^CD206^+^ (p < 0.001) macrophages were present in CIS fields and iron^+^CSF1R^+^AIF1^+^CD206^+^ populations were largely the same in the CIS and INV microenvironments (p > 0.05). Thus, we demonstrate that TAMs in the human breast cancer microenvironments differ in their phenotype, and that iron accumulation occurs in polarized TAMs of human breast cancer. Interestingly, the iron^+^ macrophages in both cancer types were associated with polarization markers as well as CSF1R, however, the fraction of these polarized iron^+^ subpopulations was biased towards M1-like and CSF1R^+^ status in the CIS regions, but also frequently exhibited mixed phenotypic character in both settings. In the context of our novel iron imaging approaches, this indicates that while macrophage polarization is an important immunological factor in both murine and human cancers, the number of iron^+^ macrophages itself can serve as a TAM imaging biomarker that is sensitive to microenvironment and stage of the cancer with high-potential for *in vivo* detection by MRI.

## Discussion

The accumulation of hemosiderin iron in tumor-associated macrophages enables the imaging of these cells in heterogeneous tumor microenvironments according to localized differences in iron metabolism. In earlier studies we reported that iron deposits occur in the stromal margins of murine mammary tumors and prostate cancers using histological imaging and *in vivo* FeMRI^23,24,40^. Recently, these iron deposits have also been detected in the stromal margins of non-small cell lung cancer tumors^37^, and we have also shown them to be present in lung and brain metastases^23^. In the current study we confirmed that such TAM iron deposits are present in multiple MMTV-PyMT breast cancer models, they are correlated with response to CSF1R breast cancer immunotherapy, and we demonstrated that the spatial infiltration and accumulation of the TAM iron deposits is a factor that is conserved in murine and human breast cancer and differentiates between human breast cancer microenvironments.

From an *in vivo* imaging standpoint our novel measurements also serve to further establish FeMRI as a unique approach for the detection and monitoring of immune cell response to cancer immunotherapy. Alternative MRI approaches label macrophages using injections of iron nanoparticles^34,35,57^, and injected radiolabelled nanoparticles used in PET imaging can also be used to measure macrophage during immunotherapy trials^8,58^. Our imaging studies are set-apart from these contrast agent-dependent techniques as we do not use contrast agent injections. We instead capitalize on the high physiological innate iron metabolism of macrophages that results in the phenotypic storage of solid deposits of iron that is readily validated using Prussian blue iron histochemistry of the TAM iron deposits. These magnetic deposits give rise to characteristic high-iron FeMRI pixel contrast that reveals their infiltration in the tumor microenvironment where they fulfill obligate roles in iron storage and recycling^21^. Although our reliance on endogenous iron stores to image TAMs is potentially restricted because we only detect those macrophages engaged in iron handling roles, our previous findings and current studies showing HLMs in the different MMTV-PyMT backgrounds as well as human cancers support the feasibility of using the iron imaging approaches to image them, and supports the combination of this form of endogenous cell-tracking with immunotherapeutic trials targeting macrophage in the clinic.

While we focus on iron as a primary biomarker of tumor macrophages in this contribution, TAMs are also recognized to adopt so-called polarization phenotypes that are closely associated with their role in immune response and communication to other immune cells such as T-cells that is critical for immunotherapeutic efficacy^48,53^. These multifactorial polarization states reflect the stage of immune response, progression of primary tumor and metastasis growth, as well as the microenvironment and tissue in which they are found^6^. We discovered that macrophage iron deposits are found more prevalently at the stromal margins of tumors in murine models and human breast cancers suggesting that iron is another microenvironmental factor influencing macrophage phenotype and spatial distribution. However, when we investigated the polarization status of the macrophage iron deposits we found them to be associated with various phenotypic polarization markers, and they were often co-localized with macrophage markers indicating mixed polarization character, rather than adopting a single polarization state. While the current studies do not address the functional significance of such polarization states in the context of their role in signaling to other immune cells, it is evident due to their clear association with iron that they serve a primary functional role in iron metabolism. In this regard it can be speculated that iron^+^ macrophages are primed to fill M1-like inflammatory functions where they sequester iron to shield it from depletion due to hemorrhage or pathogen, as well as function in M2-like wound-healing roles where they serve as stores of iron to sustain cellular proliferation of the microenvironmental milieu during tissue repair^19^. This scenario is supported by our observations of the differences in iron^+^ macrophage of *in situ* and invasive human cancers. Here, more iron^+^ macrophages were found in earlier cancer stages suggesting that these macrophages avidly stored iron during initial inflammatory immune response to the cancer. Similarly, in advanced cancers reduced numbers of iron-containing TAMs presumably reflects the depletion of stromal macrophage iron stores in order to fuel the cancer’s malignant outgrowth as it has been proposed that cancer cells have a pronounced dependence on iron metabolism that serves to co-opt this critical micronutrient from other cellular species in the tumor microenvironment^60,61^. In this context, the iron^+^ deposits can themselves be used as specific histological and *in vivo* biomarkers of TAM infiltration which varies with immune status and cancer stage without the need for assessment of tissue-dependent polarization.

Immune therapies such as the small molecule inhibitors of macrophage colony stimulating factor 1 receptor (CSF-1R) including BLZ945 and PLX3397, as well as antibodies directed against this receptor have been shown to reduce the accumulation of TAMs in preclinical models and achieve therapeutic gains as monotherapy and in combination with other therapies^48,52^. We confirmed the primary immunotherapeutic effects of the CSF1R inhibitor in the MMTV-PyMT mammary tumor models by demonstrating that the drug achieves reduction in macrophage accumulation together with tumor growth inhibition. We also confirmed that the iron^+^ macrophages express CSF1R in murine and human breast cancers supporting their role as targets of these immunotherapies. Indeed, inhibition of macrophage accumulation by CSF1R therapy resulted in significant reductions of iron-laden macrophage deposits in the orthotopic MMTV-PyMT models. Therefore, these studies validated the immunotherapeutic effect of the drug in the breast tumor models, further identified the iron-laden macrophage populations as responsive to the CSF1R immunotherapy, and confirm their CSF1R status for future therapeutic targeting in human cancer.

Cancer therapies targeting iron metabolism under current investigation have sought to either achieve therapeutic gains by iron loading using nanoparticle theranostic injections, or by causing iron depletion with iron chelators^24,37,39,62^. In the current studies we identify another means of targeting cellular iron for therapy. By capitalizing on the observation that iron deposits are restricted to TAMs, we demonstrate that CSF1R inhibition that directly targets TAMs can also be used to indirectly prevent accumulation of TAM iron deposits. As our murine investigations and surveys of human breast cancers confirm the presence of these cells and characterize their spatial dependencies, targeting the iron deposits using CSF1R inhibitors and spatially monitoring them using iron imaging has notable potential value as a translational therapeutic cancer imaging strategy. Further applications of this approach include diseases such as iron overload disorder, neurodegeneration, inflammation, and hemorrhage where iron deposits may be present and their detection, mapping, and subsequent therapeutic reduction can be desirable^41,42,59,63^.

## Conclusions

In sum we identified iron deposition as a metabolic biomarker of macrophage infiltration in murine and human breast cancer that identifies responsive polarized TAM populations to CSF1R immunotherapy using histological and *in vivo* iron imaging together with preclinical cancer research approaches. While the current studies support our ability to image polarized TAMs according to iron status in breast cancer, and suggest that iron deposits are associated with specific types of cancer, given the diverse types of human breast cancers encountered in the clinic, further histopathology will be required to more completely characterize the association of the deposits with the myriad immunological markers involved in immunotherapeutic studies, as well as correlate their accumulation with cancer stage, and clinical outcome. Also, though these findings support the translation of the FeMRI approaches to human breast cancer, and our detection of iron deposits in human cancer strongly supports the feasibility of this approach, further imaging validations will ultimately determine whether the approaches presented here in preclinical models will find their niche in clinical cancer imaging and immunotherapy trials in the future. Therefore, these demonstrations support such future studies that seek to image immune cells and harness their innate functional and metabolic dependencies for therapy.

## Methods

### Magnetic resonance imaging

MRI was performed on a 7T/30 cm horizontal bore Bruker Biospec MRI system (Bruker Biospin Corp.) with a custom-built 30 mm inner-diameter transmit-receive quadrature radio-frequency coil.

### Iron MRI

2D multi-gradient echo (MGE) relaxometry pulse sequence was used with the following parameters: TR/TE 1.2 s/3 ms × 16 echos, *in vivo* spatial resolution 0.1 mm × 0.1 mm × 1 mm, *ex vivo* 0.05 mm × 0.05 mm × 0.5 mm, RF flip angle 60°, and each spatial encoding phase encode acquisition was gated on the animal’s respiratory cycle. The first image of the gradient-echo series was used as reference images shown in the figures and overlays, and all images shown and analyzed correspond to axial mid-section slices of the tumors.

Aqueous solutions of iron(III) nitrate (Fisher Scientific) were used as standards for iron concentration measured at 7T as described in refs^23,24,40^. The T_2_* values for these solutions was determined by pixel-wise monoexponential fitting of the MGE images using Fiji^64^, and the linear relation between the relaxation rate R_2_* = 1/T_2_* and known iron concentrations was found and subsequently used to generate parametric maps of iron concentration. Quantification of high-iron FeMRI clusters was conducted by binary stratification with respect to the median of the concentration range of the iron maps followed by counting the frequency of the high-iron pixel clusters with the Fiji Analyze Cluster tool. Infiltration profiles of the high-iron clusters were generated by measuring them as a function of position according to a concentric ring pattern generated with the Fiji ROI Manager tool macros.

### Animal procedures

All animal studies were approved by the MSKCC IACUC committee and performed in accordance with their guidance and regulations.

### *In vivo* MRI

Mice were anesthetized with 1–3% isoflurane in O_2_ gas, and respiration was monitored during all imaging sessions.

### Primary tumor models

Female 6 week-old FVB/N or C57BL6 mice underwent orthotopic mammary fat pad injection of 1 × 10^6^ syngeneic TS1 MMTV-PyMT or 99LN tumor cell lines grown and collected using standard tissue culture conditions and suspended in a 50% solution of Matrigel and saline (BD Bioscience). Endpoint was defined as when the control cohorts average tumor size reached approximately 1 cm^3^ determined by caliper, MRI, or veterinary staff inspection notice.

### CSF1R inhibitor administration

The CSF1R inhibitor BLZ945 (Novartis) was administered by oral gavage (200 mg kg^−1^ in captisol vehicle, 5×/week). Dosing commenced once tumors exceeded approximately 100 mm^3^ and continued until control cohorts average tumor size reached approximately 1 cm^3^ determined by caliper, MRI, or veterinary staff inspection notice.

### Flow cytometry

Tumor-bearing mice at endpoint were perfused with 20 mL of PBS prior to mammary tumor retrieval at endpoint. Tumors were washed once in PBS and enzymatically digested for 45 minutes into a single cell suspension using a tumor dissociation kit (Miltenyi Biotec). Cell suspensions were filtered twice through 70 μm cell strainers, and Fc-blocked for 30 minutes on ice (1:50; clone 2.4G2, BD Bioscience). Cells were then immunostained using antibodies (BioLegend) for CD45 (1:400; clone: 30-F11), Ly-6C (1:400; clone HK1.4), Ly-6G (1:400; clone 1A8), CD11b (1:200; clone M1/70), and F4/80 (1:250; clone BM8) for 45  minutes on ice. Cells were then washed twice in PBS and suspended in FACS-buffer (2% FBS in PBS) containing DAPI (2.5 μg/mL; Invitrogen) for exclusion of dead cells. TAMs were identified as DAPI^−^CD45^+^Ly6C^−^Ly6G^−^CD11b^+^F4/80^+^ cells, and frequency of TAMs was determined from counts in the F4/80^+^CD11b^+^ flow cytometry gates with respect to total live CD45^+^ cells using FCS Express (De Novo Software).

### Histology

Cross-sections from the PBS-perfused MMTV-PyMT tumors were sliced at the axial tumor midpoint and fixed in 4% PFA for 24 hours at 4 °C, and then washed with H_2_O and re-suspended in 70% ethanol (Fisher Scientific). Human tissue sections donated to the Molecular Cytology Core Facility by the Pathology Department were acquired under MSKCC Institutional Review Board informed consent and provided for the study without any unique patient identifiers except diagnosis. They were fixed in 10% neutral buffered formalin. All tissues were paraffin embedded and 5 μm sections cut onto glass slides and coverslipped prior to histological quantification.

The Prussian blue histochemical detection of iron(III) was performed by standard methods using 30 minute staining of de-parrafinized slides in equal parts 10% potassium ferricyanide (Fisher Scientific) and 10% hydrochloric acid (Fisher Scientific) followed by nuclear-fast red counter-staining for 30 minutes.

The immunofluorescent detection of CD68 (Boster, cat# PA1518, 5 ug/ml, 5 hours) was performed using standard Discovery XT processor protocols and reagents (Ventana Medical Systems) with biotinylated goat anti-rabbit antibodies (Vector Labs, cat#PK6101, 60 minutes) at 1:200 dilution, and visualized with Tyramide Alexa Fluor A546 (Invitrogen, cat# T20933) and DAPI (Sigma Aldrich, cat# D9542, 5 µg/ml) prepared according to manufacturer’s instructions with predetermined dilutions.

Multiplex immunofluorescent stainings were performed as previously described^56^. Prussian blue stained slides were differentiated, and sections were then sequentially incubated with anti-CSF1R (Santa Cruz, cat#sc-692, 0.5 µg/ml, 5 hours) and biotinylated goat anti-rabbit IgG (Vector labs, cat#PK6101, 60 minutes) at 1:200 dilution, anti-CD206 (Abcam, cat#ab64693, 1 µg/ml, 5 hours) and biotinylated goat anti-rabbit IgG (Vector labs, cat#PK6101, 60 minutes) at 1:200 dilution, and finally anti-AIF1 (Wako, cat#019–19741, 0.5 µg/ml, 5 hours) and biotinylated goat anti- rabbit IgG (Vector labs, cat#PK6101, 60 minutes) at 1:200 dilution. The detection was performed successively for each of the antibodies using streptavidin-HRP D (part of DABMap kit, Ventana Medical Systems) followed by incubation with Tyramide Alexa 488 (Invitrogen, cat# B40953) for CSF1R, Tyramide Alexa 647 (Invitrogen, cat# B40958) for CD206, and Tyramide Alexa 568 (Invitrogen, cat# T20948) for AIF1, and  DAPI (Sigma Aldrich, cat# D9542, 5 µg/ml) prepared according to manufacturer instruction with predetermined dilutions.

Histological sections were digitized with a Mirax scan system and read with Panoramic Viewer (3DHISTECH). Images were first visually inspected for quality and then processed to remove background fluorescence and provide maximal signal for binary spot counting performed over whole cross-sections or selected fields from exported images using Fiji according to staining or immunofluorescent labeling. Deposits of iron containing macrophages and their infiltration profiles were quantified from the Prussian Blue histology using the Fiji Analyze Cluster tool and ROI Manager macro tools as described in refs.^23,24,40^ and also applied to the FeMRI data. Evaluation of iron^+^ macrophage polarization phenotype in murine and human tissue was conducted by co-registering Prussian blue and triple-stained immunofluorescent images, identification of 200 µm × 200 µm fields centered on iron deposits, and performing exhaustive binary counts made of the iron^+^ cells as a function of AIF, CD206 and CSF1R positivity in Fiji. Fractional populations were calculated by dividing the total counts of a given set of co-localized macrophage markers by the sum of the markers being compared, for example M2-like polarization of iron containing macrophage was calculated by dividing the iron^+^CD206^+^ population by the sum of iron^+^AIF1^+^, iron^+^CD206^+^, and iron^+^AIF1^+^CD206^+^ populations.

### Statistics

Statistical tests are indicated in the figure legends when performed and significance is determined as p < 0.05 in all analyses. All statistical analyses were performed with GraphPad Prism 7 (GraphPad Software).
